# Recognition of *Neisseria meningitidis* by the Long Pentraxin PTX3 and Its Role as an Endogenous Adjuvant

**DOI:** 10.1371/journal.pone.0120807

**Published:** 2015-03-18

**Authors:** Barbara Bottazzi, Laura Santini, Silvana Savino, Marzia M. Giuliani, Ana I. Dueñas Díez, Giuseppe Mancuso, Concetta Beninati, Marina Sironi, Sonia Valentino, Livija Deban, Cecilia Garlanda, Giuseppe Teti, Mariagrazia Pizza, Rino Rappuoli, Alberto Mantovani

**Affiliations:** 1 Department of Inflammation and Immunology, Humanitas Clinical and Research Center, Rozzano (Milan), Italy; 2 Novartis Vaccines and Diagnostics Research Center, Siena, Italy; 3 Unidad de Investigación. Hospital Clínico Universitario, Valladolid, Spain; 4 Metchnikoff Laboratory, University of Messina, Messina, Italy; 5 Humanitas University, Rozzano (Milan), Italy; University of Palermo, ITALY

## Abstract

Long pentraxin 3 (PTX3) is a non-redundant component of the humoral arm of innate immunity. The present study was designed to investigate the interaction of PTX3 with *Neisseria meningitidis*. PTX3 bound acapsular meningococcus, Neisseria-derived outer membrane vesicles (OMV) and 3 selected meningococcal antigens (GNA0667, GNA1030 and GNA2091). PTX3-recognized microbial moieties are conserved structures which fulfil essential microbial functions. *Ptx3*-deficient mice had a lower antibody response in vaccination protocols with OMV and co-administration of PTX3 increased the antibody response, particularly in *Ptx3*-deficient mice. Administration of PTX3 reduced the bacterial load in infant rats challenged with *Neisseria meningitidis*. These results suggest that PTX3 recognizes a set of conserved structures from *Neisseria meningitidis* and acts as an amplifier/endogenous adjuvant of responses to this bacterium.

## Introduction

*Neisseria meningitidis* (Nm) is a leading cause of bacterial meningitis and severe sepsis [1, 2]. This bacterium is a gram-negative encapsulated commensal organism, carried harmlessly in the nasopharynx by an estimated 10% of the world population [3]. Occasionally the bacterial pathogen can cross the oropharyngeal mucosal barrier, enter the bloodstream and cross the blood brain barrier [4], causing a devastating disease associated with mortality rates exceeding 20%. Based on the composition of the polysaccharide capsule, Nm strains can be classified in 13 different serogroups, but almost all human disease cases are caused by serogroups A, B, C, Y and W-135, and more recently by serogroup X responsible for epidemics mainly in Africa. Conventional vaccinology approaches have led to the development of a capsular polysaccharide-based vaccine against serogroups A, C, Y and W-135 [5]. In addition a vaccine against Meningococcus B (Bexsero) based on a "reverse vaccinology" approach has been recently licensed in Europe [6].

Meningococcus type B (MenB) is the major cause of invasive meningococcal disease in most countries, with incidence ranging from one case per 100,000 per year to six cases per 100,000 per year, peaking in children between age 6 months and 2 years [7]. The capacity to colonize human beings efficiently and cause high levels of bacteraemia is dependent on the ability of MenB to evade the immune system [8, 9]. MenB has developed several strategies to evade host immune responses, including the expression of a highly dynamic genome and of surface structures which mimic host molecules. In particular the meningococcus can evade killing by complement, a first line of defence against microbes [10], by expressing membrane molecules recognizing host complement inhibitors, such as Factor H (FH) [11–13]. Thus, despite the fact that the development of an effective vaccine is essential to prevent serogroup B meningococcal diseases, the challenge to eradicate MenB could also take advantage from strategies that potentiate the immune response against meningococcus, or that circumvent evasion of the immune response by meningococcus.

Fluid-phase Pattern Recognition Molecules (PRMs), including collectins, ficolines and pentraxins, are essential components of the humoral innate immune system [14]. The first soluble PRM identified was the pentraxin C-reactive protein (CRP), a main acute phase molecule in humans and the prototype of the evolutionary conserved family of pentraxins. Pentraxin 3 (PTX3) is the first member of the long pentraxin subfamily: it is characterized by a long unrelated N-terminal domain associated to the C-terminal pentraxin-like domain homologous to CRP and to the cognate molecule Serum Amyloid P Component (SAP) [15, 16].

The mature PTX3 is a complex octameric glycoprotein [17] locally and rapidly produced by a number of different cell types (e.g. monocytes/macrophages, myeloid dendritic cells, endothelial and epithelial cells) in response to primary proinflammatory stimuli (e.g. tumor necrosis factor-α, TNFα, and interleukin 1β, IL1β) and Toll-like receptor (TLR) agonists. In addition, polymorphonuclear leukocytes (PMN) store PTX3 in specific granules and promptly release the protein upon stimulation with microbial products [18].

PTX3 is a multifunctional molecule capable of interacting with several proteins, including complement components, microbial moieties and adhesion molecules [19–26]. It facilitates recognition and phagocytosis of fungal conidia mainly by PMN via Fcγ receptors (FcγRs and complement pathway mechanisms [27, 28]. In addition PTX3 can regulate inflammatory reactions dampening P-selectin dependent neutrophil recruitment at sites of inflammation [25, 29].

*Ptx3-*deficient mice are characterized by a higher susceptibility to infection with selected pathogens, such as *Aspergillus fumigatus*, *Pseudomonas aeruginosa*, *Salmonella typhymurium* and uropathogenic *Escherichia coli* [18, 30–32]. In humans, genetic evidence of the relevance of PTX3 in innate resistance has been described in pulmonary tuberculosis, in cystic fibrosis patients with *P*. *aeruginosa* lung infection and in invasive aspergillosis in patients undergoing hematopoietic stem-cell transplantation [33–35]. In addition PTX3 has a therapeutic potential in models of experimental infection with *A*. *fumigatus* and *P*. *Aeruginosa* [36–39]. Given the role of PTX3 in the orchestration of innate immunity including complement activation, essential for resistance against Nm, and its high levels in patients with meningococcal sepsis [40], we decided to investigate PTX3 interaction with Nm. Here we report that PTX3 binds MenB, recognizes selected recombinant surface proteins from MenB, and has protective activity against infection *in vivo*.

## Materials and Methods

### Ethic statement

Procedures involving animals and their care were conformed to institutional guidelines in compliance with national (4D.L. N.116, G.U., suppl. 40, 18–2–1992) and international law and policies (EEC Council Directive 86/609, OJ L 358,1,12–12–1987; NIH Guide for the Care and Use of Laboratory Animals, US National Research Council 1996). The procedures used in the present study were approved by the Animal Care and Use Committee of the Istituto Clinico Humanitas and by the Department of Pathology and Experimental Microbiology Committee for Animal Studies.

Animals were housed in the specific pathogen free Animal Facility at Istituto Clinico Humanitas. Groups of 5–7 mice were housed in individually ventilated cages with 12 hours dark/light cycle and *ad libitum* access to autoclaved food and water. A certified veterinarian is responsible for animal welfare supervision and regular health monitoring of the Animal Facility. All efforts were made to minimize the number of animals used and their suffering. Animals were euthanized by cervical dislocation after sedation.

### Cell Culture media, proteins and reagents

The following reagents were used for tissue culture: pyrogen-free saline (Baxter Italia, Milan, Italy); phosphate buffered saline (PBS) with calcium and magnesium (PBS^+/+^; Biosera, Biotecna, Milan, Italy); Hank’s Balanced Salt Solution (HBSS), RPMI 1640 and L-glutamine (Lonza, Basel, Switzerland); aseptically collected foetal calf serum (FCS; HyClone Laboratories, Logan, UT, USA). Lipopolysaccharide (LPS) from *Escherichia coli* strain 055:B5 and Bovine serum albumin (BSA) with low endotoxin characteristics were obtained from Sigma-Aldrich (Milan, Italy). Ovoalbumin for ELISA and EndoFit Ovoalbumin (endotoxin content < 1EU/mg of protein) used for immunization were from Invivogen (San Diego, CA, USA). Human C reactive Protein, Serum amyloid P component and Histone H1 from calf thymus were from Merck (Darmstadt, Germany).

Recombinant human and murine PTX3 and human C-terminal (C-PTX3) and N-terminal (N-PTX3) domains were expressed in Chinese hamster ovary (CHO) cells and purified by immunoaffinity from culture supernatants as previously described [41]. Biotinylated PTX3 (bPTX3) was obtained following standard protocols. Recombinant proteins were routinely tested for LPS contamination using the Limulus Amebocyte Lysate test for endotoxin (sensitivity of 0.05 EU/ml—Lonza) and no detectable levels of LPS were measured. A rabbit polyclonal antiserum raised against human PTX3 but recognizing also murine PTX3 was used in some binding experiments.

The following recombinant surface molecules from Nm were used in the present study: genome-derived Neisserial antigen (GNA) 0278, GNA0667, GNA1030, GNA1220, GNA1990, GNA2091, GNA1870 (factor H binding protein, fHbp), and GNA2132 (Neisserial heparin binding antigen, NHBA) [12, 42, 43]. Recombinant proteins were expressed as His- or GST-tagged molecules in *Escherichia coli* and purified as previously described [44]. Polyclonal antibodies against the recombinant purified proteins were raised in mice (GNA1994, fHbp and NHBA) or rabbits (GNA2091, GNA1030) as described previously [45].

Outer membrane vesicles (OMV) were obtained by sodium deoxycholate extraction on the whole bacteria, strains NZ98/254 or H44/76, as previously described [46].

### Bacterial strains and growth conditions

The serogroup B strain MC58 and the unencapsulated MC58 cap- knock-out mutant were used for binding experiments [47]. H44/76 strain was used to measure serum bactericidal antibody titers. Bacteria were routinely grown on GC agar (BD Biosciences, Milan, Italy) or Chocolate Agar plates at 37°C and 5% CO_2_ overnight. The serogroup B strain 2996 was used for *in vivo* experiments. Bacteria were washed twice in non-pyrogenic PBS and resuspended to the desired concentrations before injection.

### Animals

129/Sv mice were obtained from Charles River (Charles River Laboratories, Calco, Italy). *Ptx3*-deficient mice on 129/Sv background were generated by homologous recombination as described [31]. Wistar rats (5 days old) obtained from Charles River were used for the infant rat model.

### Binding assays

Binding of bPTX3 (5–500 nM considering a molecular weight of 45 kDa for the PTX3 monomer) to plastic-immobilized proteins was performed essentially as previously described [24]. For the calcium-dependency study, binding of PTX3 to OMV and to purified Neisserial antigens was performed in the presence of 10 mM ethylene glycol tetraacetic acid (EGTA). Immobilization onto plastic wells of non-recognized antigens was verified using mouse or rabbit polyclonal antibodies against selected Nm antigens. To characterize the specific binding, bPTX3 (0.56–17.92 pmol) was added to triplicate wells coated with 125 pmol/well of GNA0667, GNA1030 or GNA2091 and the amount of bound PTX3 was converted to picomolar concentration using a standard curve of bPTX3. Kd was obtained by nonlinear fitting of the saturation curves by means of GraphPad Prism 4.0a software (GraphPad, San Diego, CA). In some experiments wells were coated with human PTX3 (1 μg/well) and incubated with 1 μg/well of GNA2091, NHBA or GNA1030. Binding was revealed using polyclonal antibodies at a 1:1000 dilution [raised in mice or rabbits against the recombinant purified proteins, as described previously [48]], followed by a 1:2000 dilution of horseradish peroxidase (HRP)-linked secondary antibody (GE Healthcare, UK). As control PTX3-coated wells were incubated with buffer alone before addition of the different antibodies. Binding to immobilized proteins was evaluated as absorbance measured at 450 nm (A_450_) after addition of tetramethylbenzidine substrate (TMB; Sigma-Aldrich)

Binding of human bPTX3 to live bacteria was evaluated by flow cytometry using a FACS-Canto (BD Biosciences, Milan, Italy). Few single colonies after O/N inoculums were grown to OD_600_ = 0,2 nm, then 50 μl of bacteria were plated in 96-U-bottom well plates and incubated with different concentrations of human bPTX3 (range 2.2–2200 nM—90 min at room temperature) in FACS buffer (1% BSA in HBSS). At the end of incubation plates were spinned (3500 rpm 5 min) and supernatant was removed: this procedure was repeated twice using HBSS as washing buffer. Bacteria were then incubated 30 min. with Streptavidin-PE (BD Biosciences) 1/100 in FACS buffer. Plates were washed twice and bacteria were fixed in 1% paraformaldehyde in PBS before FACS analysis. In neutralization experiments, cells were preincubated with unlabeled PTX3 (1.1 μM) for 10 minutes at room temperature before addition of bPTX3.

### Infant rat model

Infant (5 days old) Wistar rats were inoculated ip with the indicated doses of PTX3 or vehicle, and simultaneously challenged ip with 4 x 10^4^ colony forming unit (CFU) MenB strain 2996. The pups were randomly assigned to control or experimental group, marked, and kept with the mother. To analyze bacterial survival, CFU were evaluated in blood samples obtained at 18h after infection, serially diluted and plated onto chocolate agar.

### Immunization protocols and serum bactericidal antibody assay

For immunization protocols 8- to 12-weeks-old male or female mice on 129/sv (WT) and *ptx3*-/- background were challenged intraperitoneally (ip) or intramuscularly (im) with different doses of OMV (range 0.2–0.05 μg/mice, as detailed in Table 1) in saline without any adjuvant. Immunization was repeated at day 21 and 35. The same immunization schedule was performed using OMV + PTX3 (2μg/mice). Blood samples for analysis were taken at day 49. In some experiments mice were challenged only twice (day 0 and 21) and blood was collected at day 35.

**Table 1:** List of immunization experiments.

Experiment #^(a)^	Sex	Genotype	Treatment	OMV (μg/mice)	n (OMV / OMV+PTX3	Route
**1**	M	WT	OMV±PTX3	0.5	10 / 10	ip
**2**	F	WT	OMV±PTX3	0.5	7 / 8	ip
		*ptx3-/-*	OMV±PTX3	0.5	7 / 8	
**3**	M	WT	OMV±PTX3	0.2	10 / 10	ip
**4**	M	WT	OMV±PTX3	0.05	8 / 7	ip
		*ptx3-/-*	OMV±PTX3	0.05	6 / 8	
**5**	F	WT	OMV	0.05	20	ip
		*ptx3-/-*	OMV	0.05	18	
**6**	M	WT	OMV	0.5	15	im
		*ptx3-/-*	OMV	0.5	16	
**7**	M	WT	OMV	0.05	25	im
		*ptx3-/-*	OMV	0.05	16	

Mice were immunized by ip or im treatment with different doses of OMV. In experiments n° 1 to 4, PTX3 (2 μg/mice) was administered together with OMV.

^a^ Sera were collected two weeks after the third immunization (experiment #1 to 5) or two weeks after the second immunization (experiment #6 and 7).

SBA against MenB strain H44/76 was evaluated as described [49]. Bactericidal titres were expressed as the reciprocal of the serum dilution yielding ≥ 50% bactericidal killing.

*Ptx3-/-* and WT animals were also immunized with OVA. Male animals were treated im with 100 μg/mice of EndoFitTM OVA or OVA+PTX3 (2μg/mice) diluted in saline following the same setting used with OMV. OVA specific antibodies were evaluated by ELISA on serum collected at day 49. Briefly, ELISA plates were coated with OVA (0.5 μg/ml in 15 mM Carbonate buffer pH 9.6), blocked with 5% dry milk in PBS^++^ and 0.05% Tween 20 (PBST), and incubated 1 h at room temperature with serial 1:2 dilutions of serum from immunized animals. After washing with PBST plates were incubated with HRP-linked secondary antibody (GE Healthcare, UK) for 1h at room temperature. Absorbance values were measured at 450 nm after addition of TMB.

### Statistical analysis

Data analyses were performed with GraphPad Prism 4.0a software, by unpaired or paired Student’s *t* test. To estimate the K_d_ (i.e. the equilibrium dissociation constant) binding of PTX3 to plastic immobilized proteins was analysed by nonlinear fitting using the equation of the "one site binding curve".

## Results

### 1. PTX3 binds Nm

**Fig 1:**
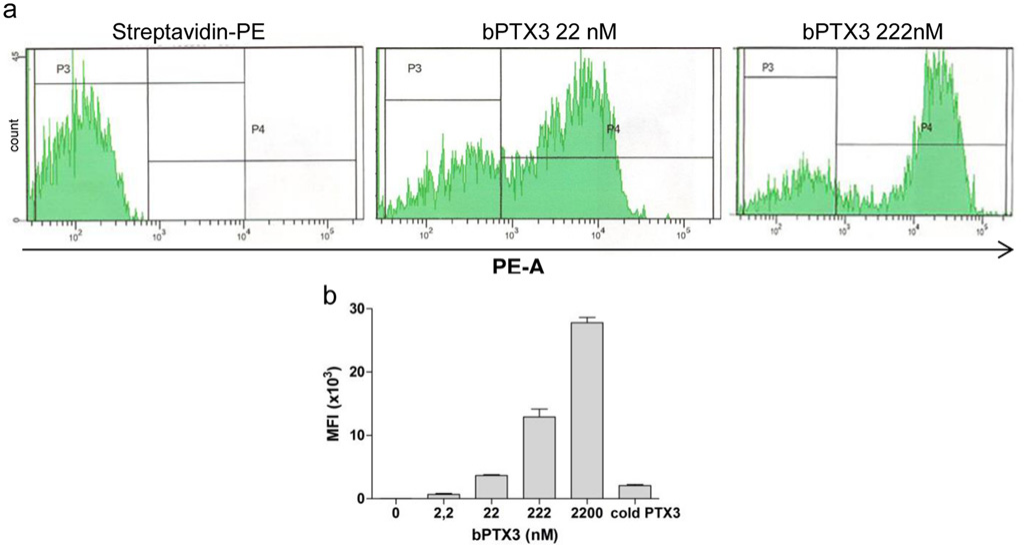
FACS analysis of PTX3 binding to *Neisseria meningitidis*. Live bacteria were incubated with different doses of human bPTX3 for 1h at room temperature. Results were assessed by flow cytometry. a) Histograms from one out of three experiments performed, showing the negative control (Streptavidin-PE) and two doses of bPTX3 (22 nM and 222 nM); b) Dose response of human bPTX3 (2.2–2200 nM) binding to Nm. Results are MFI ± SD from three independent experiments. Preincubation with cold human PTX3 (1.1 μM—10 min at room temperature) before addition of bPTX3 (222 nM) results in a 86.5% reduction of MFI.

It has been previously shown that PTX3 binds selected microbes [24, 31, 50]. In order to evaluate whether PTX3 can interact with group B Nm, we incubated human bPTX3 with live encapsulated and unencapsulated MC58 bacteria. While no binding was observed to wild type MC58 (not shown), by flow cytometry we found a dose dependent binding of bPTX3 to the mutant MC58 cap-, lacking the polysaccharidic capsule (Fig. 1). Histograms from a representative experiment are reported in Fig. 1a while Fig. 1b reports MFI ± standard deviation (SD) for the range of bPTX3 concentrations used (2.2–2200 nM). To verify the specificity of binding, bacteria were preincubated with unlabelled PTX3 (1 μM) before addition of bPTX3 (222 nM). As shown in Fig. 1b, binding of bPTX3 can be neutralized by preincubation of live bacteria with an excess of unlabelled protein.

**Fig 2:**
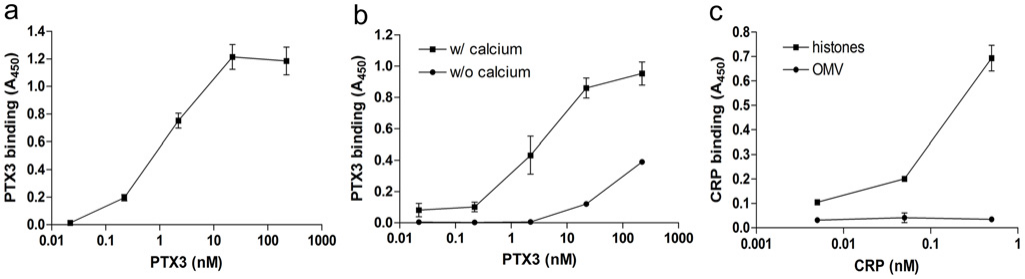
Interaction of PTX3 with OMV. Microtiter plates were coated with OMV (1 μg/well) and binding of PTX3 (a, b) or CRP (c) is presented as mean absorbance at 450 nm (A_450_) ± SD of triplicate wells. For each experimental setting data are from one out of three independent experiments. a) dose response of bPTX3; b) binding of bPTX3 to coated OMV in the presence or absence of calcium; c) Interaction of CRP with OMV. Microtiter plates were coated with 1 μg/well of OMV or Histone H1 (used as positive control for CRP binding), and incubated with different doses of CRP.

OMV prepared by detergent extraction from bacteria are characterized by a set of proteins including PorA, PorB, OpcA and NspA, and represent a tool for vaccination [51]. As shown in Fig. 2a, human bPTX3 binds OMV immobilized on plastic wells in a dose-dependent and saturable way; the interaction is calcium-dependent, as demonstrated by the reduction of binding observed in the presence of EGTA (Fig. 2b; 86% and 59% reduction for binding with bPTX3 22 and 222 nM respectively). Given the homology between PTX3 and the prototypic short pentraxin CRP, we investigated whether CRP recognizes OMV. Human CRP did not bind OMV (Fig. 2c) while, in the same experimental setting, it binds immobilized Histone H1 (1 μg/ml), a well characterized ligand of this short pentraxin used as positive control [52].

### 2. PTX3 binds selected purified recombinant membrane proteins from Nm

**Fig 3:**
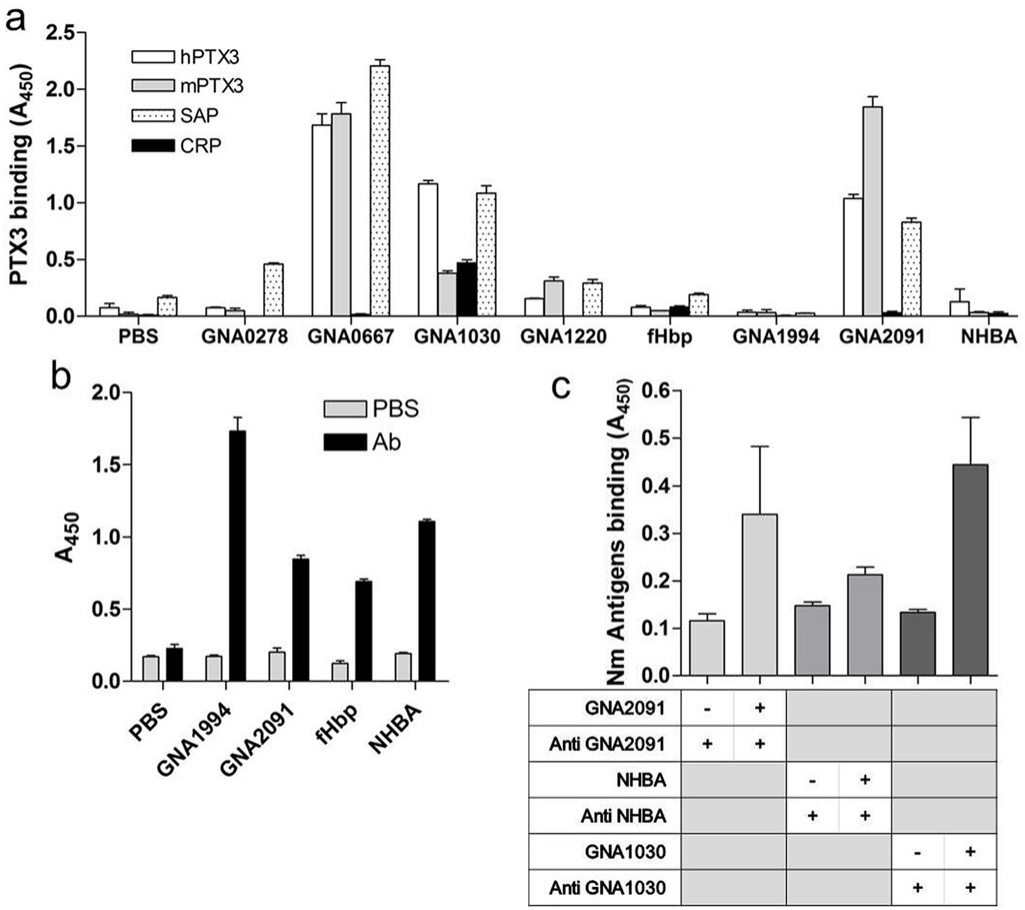
Interaction of PTX3 with recombinant proteins from *Neisseria meningitidis*. Binding was evaluated on plastic-immobilized proteins and expressed as mean A_450_ ± SD from triplicate wells. a) Plastic wells were coated with the different recombinant proteins (1 μg/well) and incubated with human or murine PTX3, human CRP and SAP (22 pmoles of all proteins) for 1 h at 37°C before addition of the different antibodies. Results are from one out of three independent experiments. b) plastic wells were coated with the indicated Nm antigens (1 μg/well) and incubated with specific polyclonal antibodies (all diluted 1:1000) against the different Nm proteins. c) Plates were coated with recombinant PTX3 (1 μg/well) and binding was evaluated incubating with the indicated Nm antigens (1 μg/well) followed by incubation with specific antibodies. As background control, incubation with Neisserial antigens was omitted while wells were incubated with the specific antibodies.

OMV vaccines, are protective only against the homologous strain. To overcome this limitation, new surface-exposed antigens able to induce bactericidal activity against different MenB strains have been identified from the genome screening of a meningococcus B strain. Further analysis identified a set of membrane antigens as potential vaccine candidates [44, 47, 53]. In order to define the molecular structures recognized by PTX3, we screened a panel of such novel genome derived antigens for binding. As summarized in Fig. 3a, human and murine PTX3 bound three different membrane proteins, namely GNA0667, GNA1030 and GNA2091, but not GNA1220, GNA0278, GNA1994, NHBA and fHbp. To verify that non-recognized molecules were immobilized on plastic wells, plates were coated with GNA1994, fHbp and NHBA and then incubated with mouse antibodies raised against the three proteins. Results reported in Fig. 3b demonstrate that GNA1994, fHbp and NHBA were indeed immobilized onto plastic wells. The interaction of PTX3 with selected proteins was confirmed also when PTX3 was immobilized on plastic wells and binding of purified Nm antigens was evaluated with specific antibodies. In this setting we observed that GNA2091, GNA1030 but not NHBA were able to bind immobilized PTX3 (Fig. 3c).

It has been reported that SAP and CRP bind Nm [54, 55]. In our experimental setting we found that SAP bound also the same molecules recognized by PTX3 (Fig. 3a); on the contrary CRP did not bind any of the proteins analysed, with the exception of a modest interaction with GNA1030 (Fig. 3a). It has been described that CRP binds in particular the phosphorylcholine (PC) moiety expressed on type 1 and 2 pili of Nm [55]. Our result is consistent with the hypothesis that PC is the main CRP ligand on the bacterial surface [56].

Thus both human and murine PTX3 recognize three conserved essential moieties of Nm which fulfil the characteristics of microbial components recognized by innate immunity (see discussion for details). Given that human and murine PTX3 have similar activities in different experimental settings (e.g. in regulation of leukocyte recruitment [25]), all subsequent experiments were performed with human PTX3.

### 3. Characterization of PTX3 interaction with proteins from Nm

**Fig 4:**
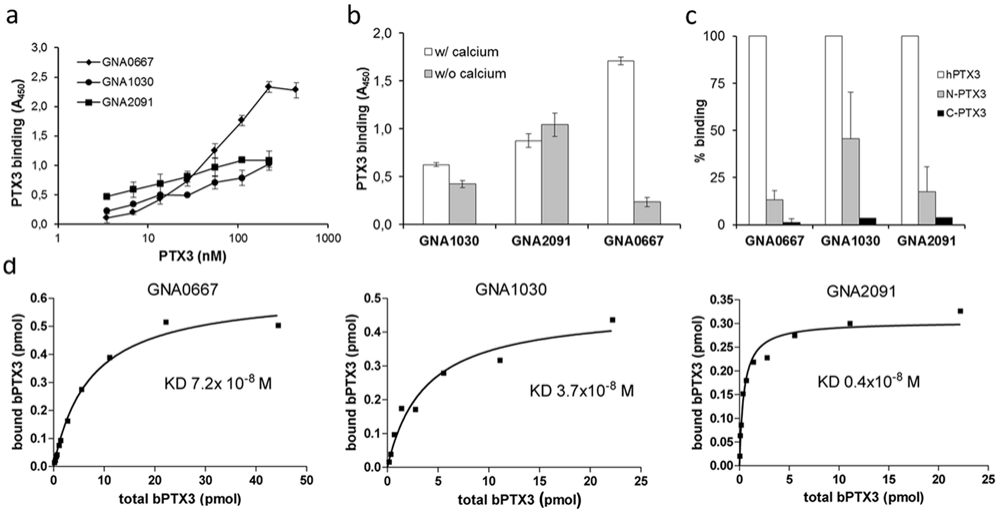
Characterization of PTX3 interaction with recombinant proteins from *Neisseria meningitidis*. a) Microtiter plate assay of the binding of different doses of bPTX3 to recombinant GNA0667, GNA1030 and GNA2091 (each used at 1 μg/well). b) Binding was performed in the presence or not of calcium. Results are from four independent experiments (mean ± SD of triplicate wells). c) Microtiter plate assay of the binding of recombinant N-terminal or C-terminal PTX3 domains (22 pmoles) to GNA0667, GNA1030 and GNA2091. Data are expressed as percentage of binding compared to recombinant full length PTX3 (mean ± SD from three independent experiments). d) Affinity of the interaction between GNA0667, GNA1030 and GNA2091, and various amount of bPTX3: specific binding was measured in accordance with a standard curve of bPTX3 with non-linear fitting analysis. Data are representative of three experiments.

The optimal PTX3 concentration for binding has been established for all the three membrane proteins in the range of 222 nM (Fig. 4a). Since calcium is required for some of the PTX3 interactions [24], we investigated the role of calcium on PTX3 binding to the recombinant antigens: as shown in Fig. 4b, calcium can affect PTX3 interaction with GNA0667 while it has no effect on the interaction of PTX3 with GNA1030 and GNA2091. We also examined the binding of Neisserial proteins to the recombinant C-terminal and N-terminal domains of PTX3 (N-PTX3 and C-PTX3) encoded by the 2^nd^ and 3^rd^ exon respectively. As shown in Fig. 4c, the recombinant C-terminal domain did not bind any of the Neisserial proteins while the N-terminal portion interacted with the three membrane proteins, though to a lesser extent in comparison to full length PTX3 (15–50% of the binding with full length PTX3; mean of three independent experiments). These results suggest that, similarly to what has been observed for other PTX3 ligands [24], the entire PTX3 molecule is required for optimal interaction with the three Neisserial proteins recognized. Finally, fitting analysis of PTX3 interaction with the three proteins has been performed (Fig. 4d) and the apparent dissociation constants have been calculated on the basis of a standard curve obtained with bPTX3. The results indicate a K_D_ equal to 3.7x10^-8^M; 0.4x10^-8^M and 7.2x10^-8^M for GNA1030, GNA2091 and GNA0667 respectively.

### 4. Amplification of antibody response by PTX3

**Fig 5:**
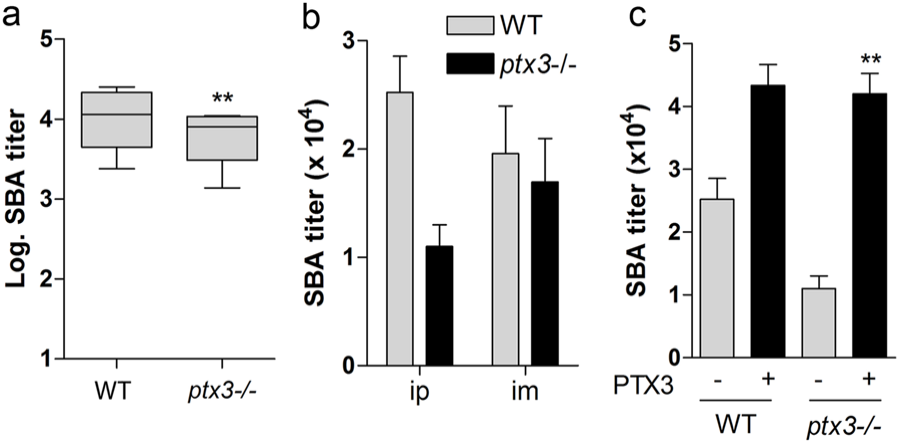
Bactericidal activity of serum from mice immunized with OMV. WT and *ptx3-/-* mice were immunized by ip or im treatment with OMV and sera were collected two weeks after the last immunization. Data are presented as mean SBA titres ± SD a) comparison of log SBA titres in WT and *ptx3-/-* mice (** p<0.01, paired Student’s t test; pooled data from ip immunized animals). b) mean SBA titres in representative experiments performed in WT and *ptx3-/-* mice immunized ip (WT n = 8 *ptx3-/-* n = 6) or im (WT n = 15, *ptx3-/-* n = 16) with 0.05 (ip) or 0.5 (im) μg OMV. c) mean SBA titres in WT and *ptx3-/-* mice immunized with 0.05 μg OMV ± 2 μg PTX3 [one out of three experiments; WT(OMV), n = 8; WT(OMV+PTX3), n = 7; *ptx3-/-*(OMV), n = 6; *ptx3-/-*(OMV+PTX3), n = 8]. *p<0.5; ** p<0.01 (Student’s *t* test).

PTX3 is one of the genes rapidly induced following treatment with adjuvants such as MF59 and CpG [57], raising the possibility that this molecule may behave as an endogenous adjuvant. To investigate this possibility we compared the response of WT and *ptx3-/-* animals in immunization protocols with OMV. Animals were immunized with OMV (0.5–0.05 μg/ml) by ip or im injection, and serum was collected two weeks after the last immunization. Seven experiments were performed with 7–20 mice per group over a period of three years (Table 1). Fig. 5a shows the pooled data from experiments 1 to 5 (ip immunization) and Fig. 5b reports two representative experiments, one for each immunization route (experiments 6 and 7). Although there was considerable variability in serum bactericidal antibody (SBA) titers within experimental groups and from experiment to experiment, *ptx3*-deficiency was associated with a significant reduction in antibody production.

The effect of exogenous administration of PTX3 in *ptx3*-competent (four experiments) and incompetent (two experiments) mice was then assessed. Fig. 5c reports the results of a typical experiment performed on WT and *ptx3*-/- mice immunized with OMV (0.05 μg/ mice, ip) ± PTX3 (2 μg/mice). PTX3 consistently increased the antibody response in *ptx3*-deficient mice, with 153 and 282% increase in SBA titres in the two experiments performed. In *ptx3*-competent mice the effect of exogenous administration of PTX3 was variable, being significant in two experiments and not significant or null in other two.

**Fig 6:**
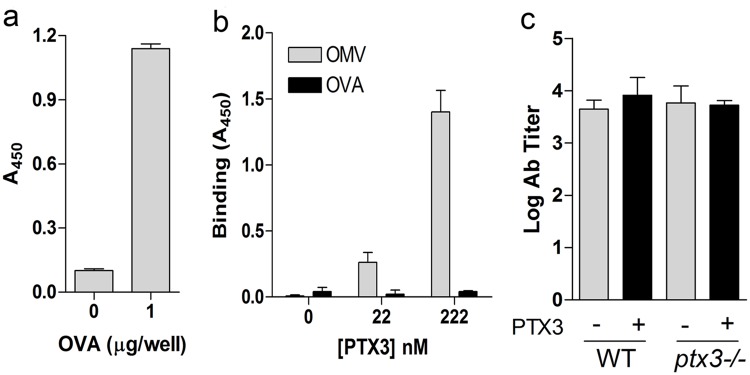
Antibody titer in mice immunized with OVA. Immune response to OVA was analyzed in WT and *ptx3-/-* mice. a) OVA was immobilized on plastic wells and presence of coated protein was confirmed by incubation with anti-OVA antibody. b) Binding to immobilized OVA or OMV as control (both at 1 μg/well) was analysed using human bPTX3. Results are reported as A_450_ (mean ± SD of triplicate wells). c) WT and *ptx3-/-* mice were immunized im with OVA (100 μg/mice ± 2 μg PTX3; each group n = 5) following the same setting used for OMV. Antibody titer was evaluated by ELISA.

The variability of the effect of exogenous PTX3 on SBA in *ptx3*-competent mice is likely a reflection of endogenous PTX3 OMV-elicited (data not shown). To verify whether the impairment in antibody production is due to a more general defect in the immune status of *ptx3-/-* animals, we investigated antibody production following immunization with a molecule not recognized by PTX3. Ovoalbumin (OVA) is a key reference protein for immunization studies, thus we first analysed whether PTX3 interacts with OVA. OVA was successfully immobilized on plastic wells, as verified with anti-OVA antibody (Fig. 6a), however in the same conditions in which PTX3 binds OMV, we did not observe interactions between PTX3 and OVA (Fig. 6b). *Ptx3-/-* and WT mice were then immunized with OVA (100 μg/mice) following the same schedule used for OMV and antibody titers were determined in serum collected two weeks after the third immunization. In contrast to what observed in the response to OMV, no differences were evident between WT and *ptx3-/-* animals in the production of anti-OVA antibodies (Fig. 6c). In addition, PTX3 administration had not effect on the antibody response elicited by immunization with OVA (Fig. 6c). Taken together these data indicate that PTX3 can potentially play a role in the response to recognized OMV but not in response to a non-recognized molecule such as OVA.

### 5. Protection against *N*. *Meningitidis*

**Fig 7:**
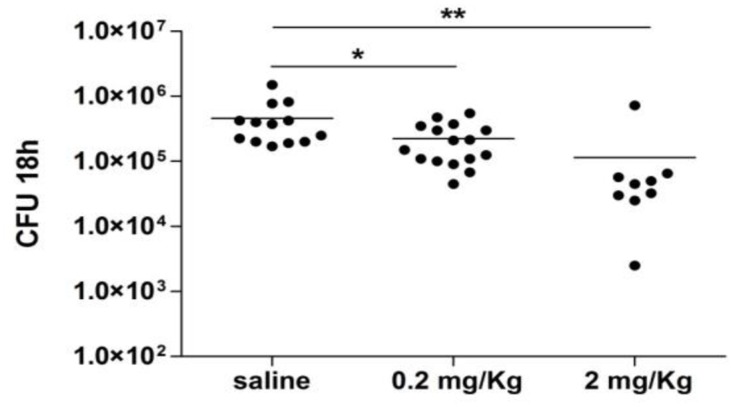
Protective effect of PTX3 in the infant rat model. Infant rats were infected ip with 4x10^4^ CFU MenB strain 2996 in association with 0.2 or 2 mg/Kg of human recombinant PTX3 (n = 16 and n = 9 respectively) or vehicle (n = 13). Blood CFU were evaluated 18 hrs after challenge. * p<0.05 (unpaired Student’s *t* test).

We then evaluated whether PTX3 can exert a protective role toward infection with MenB. Since mice are resistant to infection with Nm, we performed infection experiments in infant rats, a well described model of infection with Nm [58, 59]. PTX3 was administered at the moment of ip infection with live bacteria (4x10^4^ ip) and animals were sacrificed 18 hours after the challenge to evaluate blood CFU. As reported in Fig. 7, administration of PTX3 in association with live bacteria significantly reduced the number of CFU compared to vehicle-treated animals.

## Discussion

The long pentraxin PTX3 is an essential component of the humoral arm of innate immunity highly conserved in evolution. The molecule is a distant relative of CRP, from which differs for gene organization, cellular sources and inducing stimuli. In particular, while CRP is systemically produced by the liver in response to IL6, PTX3 is locally produced by different cell types, including myeloid, endothelial and epithelial cells, in response to primary inflammatory stimuli or following microbial recognition [14]. Gene targeting of PTX3 has unequivocally defined its role in innate immunity and inflammation, revealing a non-redundant role of this molecule in resistance to selected pathogens. In the present study, prompted by the finding of high levels of PTX3 during meningococcal infection in humans [40] and induction by adjuvants [57], we focused on the possible interaction of PTX3 with Nm.

We found that PTX3 binds to unencapsulated but not to encapsulated Nm, OMV, and three surface proteins, GNA2091, GNA1030 and GNA0667. The latter molecules were identified in a reverse vaccinology effort [44, 47] and two of them were selected as components of a universal MenB vaccine because of their conservation among strains and immunogenicity [6, 49, 53, 60]. The binding was selective, in that other structures from Nm were not recognized and the short pentraxin CRP did not interact with the three PTX3-recognized molecules. The N-terminal domain encoded by exon 2 was able to bind all three Nm molecules, but it did not fully recapitulate the binding capacity of the full molecule containing the exon 3-encoded pentraxin domain.

The polysaccharide capsule of *N*. *Meningitidis* has an ambiguous role in pathogenesis. The presence of a capsule protects the meningococcus from phagocytosis and complement-dependent killing, allowing its growth in the blood [61–63]. In agreement almost every strain recovered from the bloodstream or cerebrospinal fluids of infected individuals expresses a polysaccharide capsule. However, the capsule can hinder the surface adhesins, preventing the adherence and entry of MenB into epithelial cells of the nasopharynx, thus interfering with the initial steps of the colonization. As a matter of fact isolates obtained from healthy carriers are frequently unencapsulated. Recognition of unencapsulated MenB by locally produced PTX3 may thus be relevant for host protection in the initial steps of nasopharyngeal colonization.

Based on sequence analysis, GNA2091 is predicted to be a surface-exposed lipoprotein with a putative haemolysin function while GNA1030 is predicted to be localized in periplasm and with a putative function on quinone metabolism [60, 64]. Knock-out for the gene encoding for GNA2091 has reduced growth ability and bacteria are more susceptible to stress conditions compared to wild-type strain, suggesting a role for GNA2091 in preserving the bacterial membrane during colonization and invasive disease [48]. GNA0667 is a ligand of the scavenger receptors SR-A and MARCO [65, 66]; its C-terminus has some homology with the zipA protein from *E*. *coli*, which is involved in septum formation during cell division. In agreement deletion of GNA0667 is lethal [66]. Therefore meningococcal surface proteins bound by PTX3 are characterized by a proven or presumed essential function in the life cycle of the bacterium and by a high degree of conservation, thus fulfilling classic criteria of microbial molecules recognized by innate immunity pattern recognition molecules [14, 67–69].

Nm activates cellular innate immunity, including cytokine production. Cytokines play a crucial role in the pathophysiology of meningococcal disease and non-LPS components of *N*. *meningitidis* induce production of proinflammatory cytokines by monocytes [66, 70–72]. In agreement, Pluddemann and co-workers demonstrated that GNA0667 blocks the binding of meningococci and acetylated low density lipoproteins to SR-A, acting as TLR agonist and stimulating the MyD88-dependent secretion of cytokines such as IL-6 and TNFα [66]. In the context of PTX3 recognition of Outer membrane protein A from *Klebsiella pneumoniae* (KpOmpA), this humoral pattern recognition molecule acts as a non-redundant amplification loop in the cascade of mediators set in motion by TLR-2 dependent activation of innate immunity by KpOmpA [24]. Thus a general picture of complementarity emerges in the recognition of conserved microbial structures by cell-associated and humoral pattern recognition molecules [73].

Lack of animal models has hindered the studies on Nm infections, making impossible the analysis of PTX3 relevance in *ptx3*-deficient mice. However, under appropriate experimental conditions, some strains of Nm can multiply in the blood of infant rats [58, 59]. By means of this model we found that PTX3 administration can reduce infection burden in infant rats challenged ip with Nm, suggesting that this molecule can exert a protective effect *in vivo*. Different mechanisms involved in the innate immune response are affected by PTX3. PTX3 can opsonize pathogens, amplifying their removal by phagocytosis and promoting earlier phagosome maturation [27, 32, 39]. In addition PTX3 has a complex role in the regulation of complement, affecting directly or indirectly all three activation pathways. PTX3 activates the classical cascade when interacting with immobilized C1q [20] and regulates the alternative pathway, localizing FH recruitment and iC3b deposition on PTX3-coated surfaces [21]. Furthermore, PTX3 binds C4 binding protein (C4BP) and enhances C4BP binding to late apoptotic cells, reducing deposition of C5b-9 and thus preventing excessive local complement activation [26]. Finally PTX3 interacts with molecules involved in the lectin pathways, Ficolin-1 and Ficolin-2 [22, 74], and Mannose Binding Lectin [23], increasing complement deposition on *A*. *fumigatus* and *Candida albicans*. The complement alternative pathway particularly contributes to the innate defence against Nm, so that the pathogen has developed several strategies to evade complement activation [75]. The polysaccharidic capsule is an important determinant of complement resistance, together with lipooligosaccharide, sialic acid, fHbp and NspA exposed on the surface of the pathogen. In particular fHbp and NspA directly bind FH thus limiting C3 deposition on the surface and enhancing resistance to complement activation. Thus it is tempting to speculate that PTX3 could either prevent binding of FH to Nm surface, allowing C3 deposition and complement activation, or could localize molecules of the lectin pathways increasing complement deposition on the pathogen. As a matter of fact, complement has a relevant role in preventing development of meningococcal disease, as evidenced by the increased incidence and recurrence of infection and disease in people with immune disorders related to FH [76, 77], or with congenital deficiencies in properdin or in one of the terminal complement components (C6, C7, C8, or C9) [76, 78, 79]. These data suggest that the interplay of PTX3 with the complement system may play a relevant role in the control of MenB infections. Thus complement is likely a key component of the PTX3-mediated innate response against Nm.

PTX3 emerged as a strongly induced gene at sites of adjuvant injection [57]. This finding, together with the amplification of Nm elicited inflammation reported here, prompted us to test the hypothesis that PTX3 acted as an endogenous adjuvant. *Ptx3*-deficiency was associated with lower antibody production (17–56% reduction, with considerable experiment-to-experiment variability). Administration of PTX3 strongly augmented antibody production in *ptx3-/-* mice (153–281%) and, less consistently in *ptx3*-competent mice. Interestingly, PTX3 adjuvant effect has not been observed when animals were immunized with a non-recognized molecule such as OVA. Thus, the humoral pattern recognition molecule PTX3, an “ante-antibody”, can amplify induction of an effective adaptive antibody response induced by a recognized antigens.

The results presented here demonstrate that PTX3 is part of innate immune response elicited by *Neisseria meningitidis*. The innate response to Nm involves activation of the cellular MyD88 and TLR dependent arm of innate immunity as well as production of a component (PTX3) of the humoral arm, with complementary functions. Coordinate and complementary activation of the cellular and humoral arm emerges as a recurrent theme in the *modus operandi* of innate immunity [24]. The potential of PTX3 as a correlate of vaccination strategies and clinical evolution as well as, possibly, as a therapeutic agent deserves further studies.
